# Aerobic exercise improves inflammation and insulin resistance in skeletal muscle by regulating miR-221-3p via JAK/STAT signaling pathway

**DOI:** 10.3389/fphys.2025.1534911

**Published:** 2025-02-25

**Authors:** Nan Li, Liang Zhang, Qiaofeng Guo, Haiyan Shi, Yanming Gan, Weiqing Wang, Xiaoying Yang, Yue Zhou

**Affiliations:** ^1^ Center for Physical Education, Xi’an Jiaotong University, Xi’an, China; ^2^ Department of Exercise Physiology, Beijing Sport University, Beijing, China; ^3^ School of Strength and Conditioning Training, Beijing Sport University, Beijing, China; ^4^ National Institute of Sports Medicine, General Administration of Sport of China, Beijing, China; ^5^ Key Laboratory of Physical Fitness and Exercise, Ministry of Education Beijing Sport University, Beijing, China

**Keywords:** aerobic exercise, skeletal muscle, insulin resistance, macrophage polarization, miR-221-3p

## Abstract

**Background:**

Exercise improves insulin sensitivity and lipid metabolism while the mechanisms remain unclear. MicroRNAs (miRNAs) have been linked to the development of type 2 diabetes mellitus (T2DM) and served as a potential therapeutic target. The study aimed to explore how aerobic exercise prevents chronic inflammation and insulin resistance (IR) in skeletal muscle.

**Methods:**

Fifty C57BL/6J male mice were divided into a normal (CON) or high-fat diet (HFD) for 12 weeks, followed by treadmill training for 8 weeks. Glucose levels were evaluated by glucose tolerance test, insulin tolerance test and kits. Chronic inflammatory states were evaluated by enzyme-linked immunosorbent assay and immunofluorescence stain. The role of miR-221-3p was determined using miRNA sequencing and dual luciferase reporter gene assays. Metabolic alterations in skeletal muscle were investigated by Real-time PCR and Western blot.

**Results:**

Aerobic exercise reduced body weight, fasting blood glucose gain, and improved insulin sensitivity. It suppressed inflammation by altering IL-1β, IL-10 levels, and macrophage polarization in the skeletal muscle. Moreover, exercise prevented chronic inflammation by diminished miR-221-3p and downstream JAK/STAT pathways.

**Conclusion:**

Aerobic exercise improved chronic inflammation and IR in the skeletal muscle, with miR-221-3p as a key modulator of macrophage polarization.

## 1 Introduction

Insulin resistance (IR) refers to a physiological condition in which peripheral tissues, such as skeletal muscle, liver, and adipose tissues, are insensitive to insulin action, leading to impaired glucose utilization and type 2 diabetes mellitus (T2DM) (Chatterjee et al., 2017). Chronic inflammation confers a significant risk for chronic diseases such as IR, T2DM, atherosclerosis, cardiovascular disease, and cancer (Rohm et al., 2022). Skeletal muscle, as a major target tissue of insulin, plays an important role in energy balance and metabolic homeostasis (Williams et al., 2020). Obesity-associated IR is related to the increased infiltration of macrophages into the inflammation of skeletal muscle (Chazaud, 2020; Wu and Ballantyne, 2017). However, the pathogenesis of IR is intricate and multifaceted, and the mechanisms of macrophage polarization in obesity-related skeletal muscle IR have not been fully elucidated.

microRNAs (miRNAs), a class of small non-coding RNAs, regulate specific gene expression through post-transcriptional and are involved in the pathogenesis of many diseases (Deiuliis, 2016). Dysregulation of miRNAs has been associated with impaired pancreatic beta cell function and reduced insulin sensitivity (Yang et al., 2016; Agbu and Carthew, 2021). Notably, miR-221-3p has emerged as a significant player in the regulation of glucose and lipid homeostasis. Overexpression of miR-221-3p has been demonstrated to induce hepatic insulin resistance by suppressing the PI3K/AKT signaling pathway (Huang et al., 2021; Huang et al., 2019). Moreover, a recent study has highlighted the upregulation of miR-221-3p in epididymal white adipose tissue of HFD mice, and targeted inhibition of miR-221 has shown the capacity to impede adipogenesis (Yamaguchi et al., 2022). It seems reasonable to presume that miR-221-3p may be a novel target for improving IR. Furthermore, as one of the target mRNAs, suppressor of cytokine signaling 1(*Socs1*) is a key part of obesity-induced inflammation and plays a critical role in IR (Olefsky and Glass, 2010; Haeusler et al., 2018). The regulation of macrophage polarization involves complex interactions with SOCS1 and miR-221-3p. Upregulation of SOCS1 promotes M2 macrophage polarization, which is associated with anti-inflammatory responses. Conversely, SOCS1 knockdown reduces Arginase 1 (Arg-1) expression and increases inducible nitric oxide synthase (iNOS) levels, lead to a pro-inflammatory environment (Whyte et al., 2011). Additionally, miR-221-3p drives M1 macrophage polarization and inflammation by targeting the SOCS1/STAT1/STAT3 signaling axis (Cai et al., 2020). As a result, inhibiting miR-221-3p has the potential to reduce inflammation and improve insulin sensitivity by modulating SOCS1. However, whether this mechanism also plays a role in chronic inflammation and insulin resistance within skeletal muscle remains unclear.

Regular physical exercise can prevent chronic diseases by improving insulin sensitivity and promoting lipid metabolism through enhanced lipolysis, fatty acid oxidation, and increased mitochondrial biogenesis (Di Meo et al., 2017; Yaribeygi et al., 2019). It has been shown that exercise-induced miR-19b-3p regulates skeletal muscle glucose metabolism by targeting and downregulating RNF11, whose silencing potentiates glucose uptake and links to Akt signaling, thereby playing a crucial role in glucose metabolism regulation (Massart et al., 2021). Exercise can also protect against IR by inhibiting MALAT1 and elevating miR-382-3p to repress resistin (Liu et al., 2019). High-glucose conditions were associated with increased levels of interleukin-1β (IL-1β) and iNOS in serum and tissue, however, exercise countered these effects by mitigating inflammation and restraining M1 macrophage polarization (Luo et al., 2021). Our previous work showed that aerobic exercise can effectively improve inflammation and insulin sensitivity in skeletal muscle (Li et al., 2022). Nevertheless, scant attention has been devoted to exploring the intricate interplay of miR-221-3p and macrophage polarization within skeletal muscle. The underlying mechanism by which exercise modulates macrophage polarization in skeletal muscle remains enigmatic. In this study, we aim to investigate the effects of miR-221-3p on aerobic exercise-induced IR and illuminate whether miR-221-3p/*Socs1* axis plays a role in regulating macrophage polarization and thus provides a theoretical basis for the treatment of IR.

## 2 Methods

### 2.1 Animals and diets

Five-week-old male C57BL/6J mice (n = 50) were obtained from Beijing Huafukang Laboratory Animal Technology Co., Ltd. (Beijing, China). The mice were housed on a reverse 12:12-h dark-light cycle at 22°C–25°C with free access to food and water. All procedures were approved by Sport Science Experiment Ethics Committee of Beijing Sport University (approval number: 2022023H).

**FIGURE 1:**
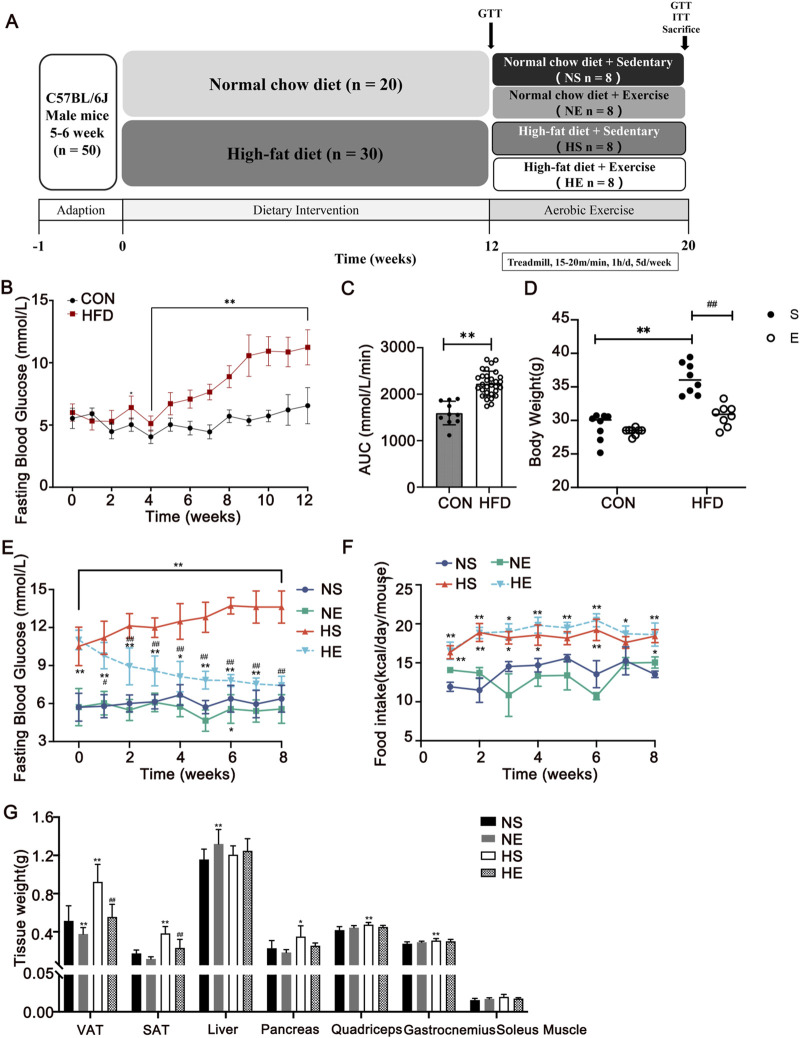
Aerobic exercise attenuated HFD-induced adiposity. **(A)** Experimental design of the study. **(B, C)** Changes in fasting blood glucose during the 12-week diet and area under the curve (AUC) between CON and HFD mice. **(D)** Changes in body weight after 8 weeks of exercise, respectively. Changes in fasting blood glucose **(E)** and food intake **(F)** during the 8-week exercise. **(G)** Tissue weights of the mice. CON, normal control, n = 20; HFD, high-fat diet, n = 30. NS, normal sedentary; NE, normal + exercise; HS, HFD sedentary; HE, HFD + exercise. VAT, visceral adipose tissue. SAT, subcutaneous adipose tissue. Values are means ± SD (n = 8 per group). *P < 0.05, **P < 0.01 vs NS group; ^#^ *P* < 0.05, ^##^ *P* < 0.01 vs HS group.

After 1-week acclimation, the mice were randomly divided into two groups: the normal control group (CON, n = 20) fed with regular chow diet (3.87 kcal/g, 12.79% energy as fat, 1,025, Huafukang, Beijing, China) and the high-fat diet (HFD, n = 30) group was fed with HFD (5.24 kcal/g, 60% energy as fat, H10060, Huafukang, Beijing, China). IR was diagnosed if the area under the curve (AUC) during glucose tolerance test (GTT) of the HFD group was 1.2 times higher than that of the CON group. After 12 weeks of diet, the mice were allocated into the following four groups: normal sedentary (NS, n = 8), normal exercise (NE, n = 8), HFD sedentary (HS, n = 8), and HFD exercise (HE, n = 8). The body weight was measured once a week using weighing scale. Food intake was measured twice a week by weighing the food provided and remaining in each cage. The consumed amount was calculated by multiplying by the caloric content to determine the weekly caloric intake. Fasting blood glucose levels were measured weekly from the tail tip after a 12-hour fasting period. (Figure 1A).

### 2.2 Exercise protocol

The exercise groups underwent acclimation for 3 days (10–15 m/min, 20–40 min/d) consecutively before beginning the exercise training sessions. Adhering to the established treadmill exercise model (Hoydal et al., 2007), the mice performed on a treadmill with a progressive increase in running speed: 15–20 m/min, 60 min/d, 5 days/week for 8 weeks. Mice in the sedentary groups were placed on the treadmill for the same duration as those in the exercise group.

### 2.3 Glucose tolerance test and insulin tolerance test

The mice underwent a glucose tolerance test (GTT) 48 h after the completion of the exercise protocol, following an overnight fast. The insulin tolerance test (ITT) was then performed on the third day after the exercise protocol, following a 4-hour fasting period. Blood glucose concentrations were measured by a glucometer (Roche Ltd., Basel, Switzerland). GTT was measured after an intraperitoneal injection of glucose (2 g/kg). To perform the ITT, calculated the volume of insulin to be injected into each mouse based on a dose of 0.5 U/kg of body weight. Blood glucose levels were measured from the tail vein at 0, 30, 60, 90, and 120 min after the glucose challenge (Vinué and González-Navarro, 2015). Homeostasis model assessment of insulin resistance (HOMA-IR) was calculated using the following equation: HOMA-IR = [Fasting Insulin (mU/L) × Fasting Glucose (mmol/L)]/22.5. HOMA-IR was calculated to assess IR, with a normal range of 1–3 in healthy, non-obese mice (Lee et al., 2008).

### 2.4 Tissue extraction

The mice were euthanized by isoflurane inhalation 48 h after the final training session. Blood samples were procured after eyeball removal, and the entirety of the blood samples were allowed to clot undisturbed at room temperature for 30 min. Blood samples were centrifuged at 4°C for 15 min at 3,000 rpm (Eppendorf 5424, rotor F-45-24-11, 5424000014). Then, only the left gastrocnemius was dissected and rapidly frozen with liquid nitrogen and stored at −80°C for biochemical analyses (n = 6 per group). For immunofluorescence experiments, the freshly excised right gastrocnemius (n = 3 per group) was covered with optimum cutting temperature compound and submerged into precooled isopentane in liquid nitrogen for 30 s. The quadriceps muscles were dissected and stored at −80°C for Western blotting and RNA analysis (n = 3 per group).

### 2.5 Immunofluorescence

Frozen muscle tissues were sectioned at 8 μm in thickness using a sliding cryostat microtome (Leica CM 1850; Germany). Three tissue sections were selected from each sample (n = 3/group). The cryosections were fixed with a 4% paraformaldehyde fixing solution for 10 min and then washed in PBS three times, permeabilized in 0.1% Triton X-100 for 15 min, and blocked with 5% normal goat serum for 1 h at room temperature. Then the sections were incubated overnight at 4°C with primary antibodies. The dilutions of primary antibodies were as follows: F4/80 (1:100, MCA497GA, Bio-Rad), CD11c (1:200, 173420-1-AP, Proteintech), CD206 (1:200, AF2535, R&D Systems). The sections were protected from light and incubated with secondary Alexa Fluor 488- or Alexa Fluor 555-labeled goat anti-rabbit or mouse antibodies (1:1,000, A-11034, A-21422, Molecular Probes) for 2 h. Nuclei were stained by DAPI. Results were observed with a fluorescence microscope (Leica, Germany). The fluorescence index was determined with Image Pro Plus 6.0 (Media Cybernetics, United States).

### 2.6 Biochemical analyses in serum and skeletal muscle

Four biochemical indicators of blood lipids were measured with kits (Njjcbio, Nanjing, China) using a clinical chemistry analyzer (AU5800, Beckman). IL-1β and IL-10 levels were measured using ELISA kits (AD3364Mo, AD2837Mo, Andygene). Procedure was followed according to the manufacturer’s instructions. Serum and tissue samples were homogenized in PBS with proteinase inhibitors, then centrifuged to collect the supernatant. Protein concentration was determined using the BCA Assay Kit. The samples were incubated in 96-well plates coated with capture antibodies, followed by detection with biotinylated antibodies and a substrate reaction. Absorbance at 450 nm was measured to determine cytokine concentrations using a standard curve.

### 2.7 Western blotting

Total proteins were isolated from skeletal muscle using RIPA protein buffer (89900, Thermo Scientific) and protease inhibitor cocktails (P8340, Sigma-Aldrich). Protein concentrations were detected using the bicinchoninic acid (BCA) protein assay kit (23227, Thermo Scientific). The samples were boiled with a loading buffer at 98°C for 10 min. The proteins were electrophoresed on 8%–12% SDS-polyacrylamide gels for separation, chosen based on target protein sizes, transferred to polyvinylidene difluoride (PVDF) membranes (88518, Cell Signaling), and blocked in 5% bovine serum albumin (BSA, A7906, Sigma-Aldrich) for 2 h. The proteins were transferred to PVDF membranes at 60 V for 1 h at 4°C. Primary antibodies (Table 1) were diluted in blocking buffer and incubated with the membranes overnight at 4°C. Then, the membranes were washed five times for 3 min in Tris-buffer saline (TBS, T1503, Sigma-Aldrich) containing 0.05% Tween 20 (TBST) and incubated with a secondary antibody conjugated to HRP for 1 h at ambient temperature. After the incubation, the membranes were washed five times for 3 min each using TBST. The density of each band was quantified using Image Lab densitometry software (Bio-Rad, Hercules, CA), and protein abundance was quantified by ImageJ. GAPDH served as a loading control to facilitate value normalization.

**TABLE 1:** Primary antibodies.

Antibody	Dilution	Catalog number	Host species	Supplier
SOCS1	1:1,000	3950	Rabbit	Cell signaling technology
JAK1	1:400	3344	Rabbit	Cell signaling technology
STAT1	1:2000	9172	Rabbit	Cell signaling technology
p-STAT1	1:1,000	7649	Rabbit	Cell signaling technology
STAT3	1:4,000	9139	Rabbit	Cell signaling technology
p-STAT3	1:1,000	9145	Rabbit	Cell signaling technology

### 2.8 miRNA sequencing

RNA was isolated from the quadriceps muscle using TRIzol. miRNAs sequence was sequenced by Sangon (Shanghai, China) using the Illumina sequencing platform (Illumina, San Diego, CA, United States). Briefly, the quality of raw sequencing reads was processed using FastQC and trimmed using CutAdapt and Trimmomatic to remove low-quality reads. Parameters for read quality filtering included sequence length >50 bp, average GC content between 30% and 70%, and a minimum Phred score of 30 for each read. Subsequently, the miRNA reads were aligned against established mature murine microRNA sequences using Bowtie (version 1.1.1). Before statistical analysis, all miRNA read counts were normalized and counted using the reads per million (RPM) method. Differential expression was performed using edgeR software and calculated using DESeq2. The threshold for DEGs was an |log2 (FC)| ≥ 1.0 and a P-value ≤0.05.

### 2.9 Quantitative reverse transcriptase-polymerase chain reaction (RT-PCR) analysis

Total RNA was extracted from the quadriceps to the manufacturer’s instructions (B511321-0100, Dalian Baosheng). Conventional cDNA synthesis was performed using RevertAid Premium Reverse Transcriptase (EP0733, Thermo Fisher Scientific). qPCR reaction was carried out in 10 μL reactions using 2X SG Fast qPCR Master Mix (B639271, Dalian Baosheng). Real-time PCR of miRNA was performed using miDETECT A Track™ miRNA qRT-PCR Starter Kit (C10712, RIBOBIO). The data presented correspond to the mean of 2^−ΔΔCT^ from at least three independent experiments after being normalized to β-actin or U6. The primers were used in Table 2.

**TABLE 2:** Primers sequences.

Gene	Primer sequences (5′-3′)	
*Socs1*	Forward Primer	CTGCGGCTTCTATTGGGGAC
	Reverse Primer	AAAAGGCAGTCGAAGGTCTCG
β-actin	Forward Primer	GTGCTATGTTGCTCTAGACTTCG
	Reverse Primer	ATGCCACAGGATTCCATACC
miR-221-3p		ACACTCCAGCTGGGAGCTACATTGTCTGC
U6	Forward Primer	CTCGCTTCGGCAGCACA
	Reverse Primer	AACGCTTCACGAATTTGCGT

### 2.10 miR-221-3p target gene prediction and validation

MiR-221-3p target gene prediction was conducted with TargetScan Mouse 7.2. To validate the interaction between miR-221-3p and Socs1, a luciferase reporter assay was performed. The 3′untranslated regions (UTRs) of *Socs1*, which contain potential miR-221-3p binding sites, were cloned into the downstream region of the Renilla luciferase gene. Mutations were introduced in the binding sites of the 3′UTR to disrupt miR-221-3p binding, and these mutated constructs were used to assess the specificity of miR-221-3p binding. The two reporter plasmids (wild-type (WT) and mutated (Mut)) were co-transfected into HEK293T cells with miR-221-3p overexpression plasmid or a negative control (NC) plasmid. Post 24 h of co-transfection, the Renilla luciferase activity was measured using the Dual-Glo luciferase reporter assay system (E2920, Promega, Madison, WI, United States) according to the manufacturer’s instructions. For controls, a luciferase assay kit negative control (NC plasmid) was used to ensure the specificity of the luciferase activity measurement. The luciferase activities of the reporter plasmids were normalized to those of the Firefly luciferase activity to account for transfection efficiency.

### 2.11 Statistical analysis

To assess statistical differences, the repeated measures analysis of variance (ANOVA) was analyzed by Bonferroni *post hoc* analysis for pairwise comparisons within the groups for body weight, food intake, and fasting blood glucose. The statistical significance of differences among groups was examined by two-way ANOVA with Bonferroni’s multiple-comparison test. For correlation analysis, the Pearson correlation test was employed. All data using Prism8 software (GraphPad software v8.0, Prism, La Jolla, CA). Values were expressed as mean ± SD and statistical significance was tested at *P* < 0.05.

## 3 Results

### 3.1 Exercise attenuated HFD-induced weight gain and dysregulation of blood glucose

To explore the contribution of a high-fat diet to the development of obesity and hyperglycemia, several parameters including body weight, fasting blood glucose, and food intake were meticulously measured over 12 weeks of intervention. Following a span of 3 weeks on the HFD, a significant rise in body weight and fasting blood glucose was evident, as compared to the normal control (CON) group (Figure 1B). Notably, the HFD group exhibited a notably larger area under the curve (AUC) in the glucose tolerance test (GTT) than the CON group (*P* < 0.01, Figure 1C). Upon IR model was confirmed, 8 weeks of exercise training was implemented in exercise groups. A substantial reduction in body weight was observed when contrasted with the CON group (*P* < 0.01, Figure 1D). This weight reduction was predominantly concentrated within visceral adipose tissue (VAT) and subcutaneous adipose tissue (SAT) (Figure 1G). Moreover, aerobic exercise reversed HFD diet-induced hyperglycemia (*P* < 0.01, Figure 1E), while the reduced caloric intake was not significant in the HE group compared with the HS group (Figure 1F).

### 3.2 Exercise ameliorated HFD-induced dysglycemia and dyslipidemia

**FIGURE 2:**
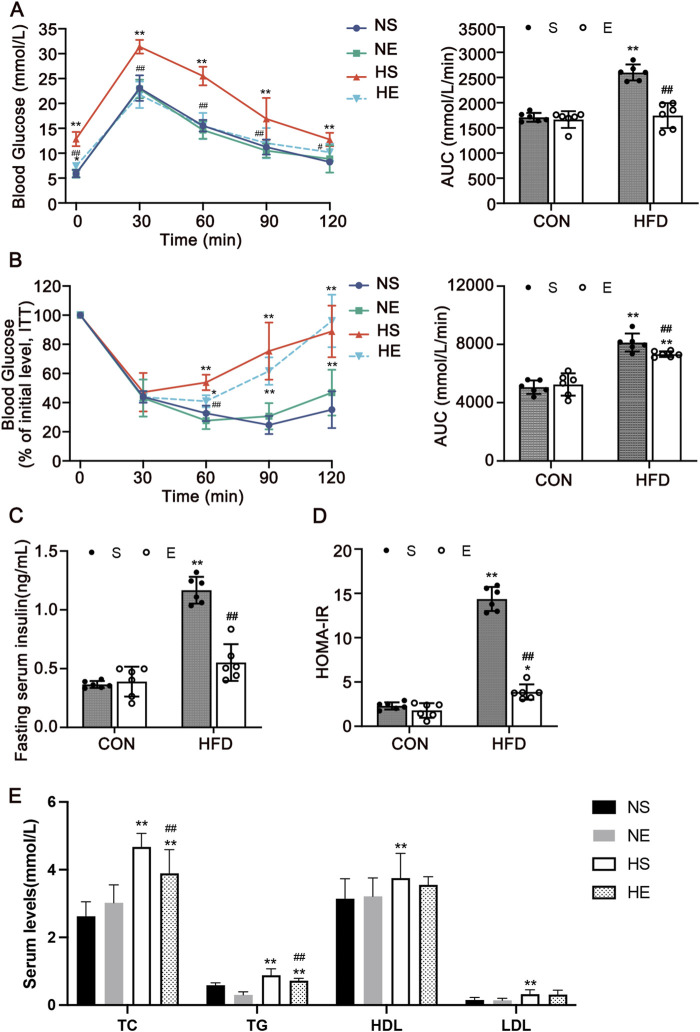
Aerobic exercise attenuated HFD-induced insulin resistance and dyslipidemia. **(A)** Glucose tolerance test (GTT) and the AUC. **(B)** Insulin tolerance test (ITT) and AUC. **(C)** Serum insulin level of mice fasted for 12 h. **(D)** Homeostatic Model Assessment for Insulin Resistance (HOMA-IR). **(E)** The levels indicators of blood lipids. Total cholesterol (TC), triglyceride (TG), high-density lipoprotein cholesterol (HDL), and low-density lipoprotein cholesterol (LDL). CON, normal control; HFD, high-fat diet; NS, normal sedentary; NE, normal + exercise; HS, HFD sedentary; HE, HFD + exercise. Values are means ± SD (n = 6 per group). *P < 0.05, **P < 0.01 vs NS group; ^#^ *P* < 0.05, ^##^ *P* < 0.01 vs HS group.

To confirm that aerobic exercise can suppress dysglycemia and dyslipidemia, GTT, Insulin tolerance test (ITT), fasting insulin levels, and blood lipid levels were measured. The GTT and ITT demonstrated that exercise improved glucose tolerance and insulin tolerance in the HE group as compared with the HS group, which was indicated by a significantly decreased AUC (*P* < 0.01, Figures 2A,B). Serum insulin levels and HOMA-IR index were also significantly increased in the HS group, which is significantly higher than the normal range, indicating the occurrence of insulin resistance. Nonetheless, this perturbation was effectively reversed following an 8-week regimen of aerobic exercise (*P* < 0.01, Figures 2C,D). Next, we examined serum levels of lipids and observed that the contents of four measures were remarkedly increased. Most importantly, the content of total cholesterol (TC) and triglyceride (TG) decreased significantly with exercise treatment (*P* < 0.01, Figure 2E). These findings suggest that aerobic exercise intervention could ameliorate dyslipidemia and dysglycemia.

### 3.3 Exercise improved HFD-induced inflammation in skeletal muscle

**FIGURE 3:**
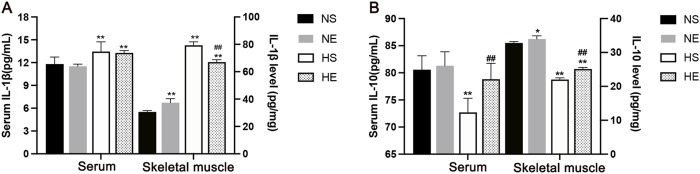
Aerobic exercise attenuated inflammation in IR Mice. **(A)** IL-1β levels in serum and skeletal muscle by ELISA. **(B)** IL-10 levels. NS, normal sedentary; NE, normal + exercise; HS, HFD sedentary; HE, HFD + exercise. Values are means ± SD (n = 3/group). *P < 0.05, **P < 0.01 vs NS group; ^#^ *P* < 0.05, ^##^ *P* < 0.01 vs HS group.

To investigate the effects of aerobic exercise on improving inflammatory status, the inflammatory cytokines IL-1β and IL-10 levels were assayed in both serum and skeletal muscle through employment of an ELISA kit. Levels of IL-1β in both serum and skeletal muscle exhibited a substantial increase as compared to the CON group. Remarkably, aerobic exercise precipitated a notable reduction in skeletal muscle IL-1β levels (*P* < 0.01, Figure 3A). Meanwhile, the M2 marker IL-10 was markedly reduced both in serum and skeletal muscle, and this effect was reversed by aerobic exercise training (*P* < 0.01, Figure 3B).

**FIGURE 4:**
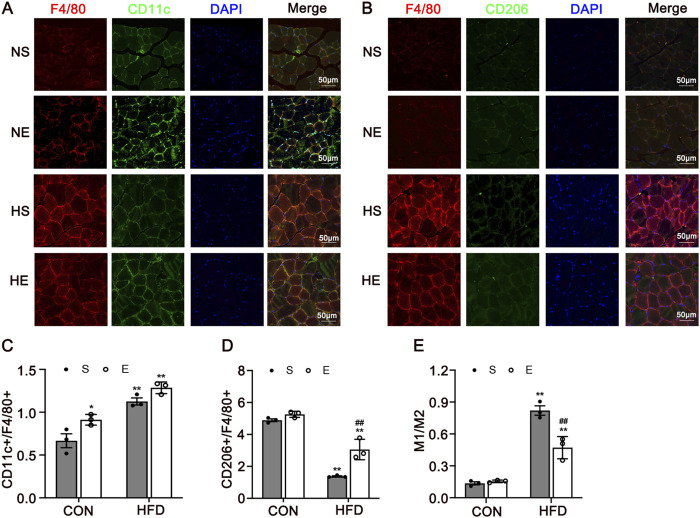
Immunofluorescence Analysis of CD11c and CD206 in Skeletal Muscle. **(A)** Representative immunofluorescence images of skeletal muscle double labeled for F4/80 and CD11c in mice. Red, green, and blue indicate F4/80, CD11c, and DAPI staining, respectively. Merged pictures show the co-localization of F4/80 and CD11c. **(B)** Representative immunofluorescence images of skeletal muscle double labeled for F4/80 and CD206 in mice. Red, green, and blue indicate F4/80, CD206, and DAPI staining, respectively. Merged pictures show the co-localization of F4/80 and CD206. Scale bar = 50 μm. **(C)** The percentage of CD11c in all macrophages in skeletal muscle. **(D)** The percentage of CD206 in all macrophages in skeletal muscle. **(E)** The percentages of M1/M2. CON, normal control; HFD, high-fat diet; NS, normal sedentary; NE, normal + exercise; HS, HFD sedentary; HE, HFD + exercise. Values are means ± SD (n = 3/group). *P < 0.05, **P < 0.01 vs NS group; ^#^ *P* < 0.05, ^##^ *P* < 0.01 vs HS group.

Macrophages are key cells in chronic inflammation, to test the phenotype of macrophages at the site of inflammation in skeletal muscle, we measured F4/80, CD11c, and CD206 protein using immunofluorescence, and counted the relative number of macrophages in each population. Skeletal muscle tissue was stained for macrophages (F4/80) and CD11c, marking M1-positive cells (Figure 4A), and for F4/80 with CD206, indicating M2-positive cells (Figure 4B). There was a significantly greater percentage of M1-type macrophages in skeletal muscle from mice fed with an HFD when compared to the CON group (*P* < 0.01, Figure 4C). Interestingly, aerobic exercise significantly increased the proportion of M2 macrophages when compared to the HS group (*P* < 0.01, Figure 4D). Further, the percentages of M1/M2 were remarkedly reduced after exercise training (*P* < 0.01, Figure 4E). Thus, HFD-induced IR is associated with increased macrophage infiltration and the chronic inflammation of skeletal muscle. Obesity-associated IR is related to the increased infiltration of phenotype of the macrophages.

### 3.4 S*ocs1* is a direct target gene of miR-221-3p

**FIGURE 5:**
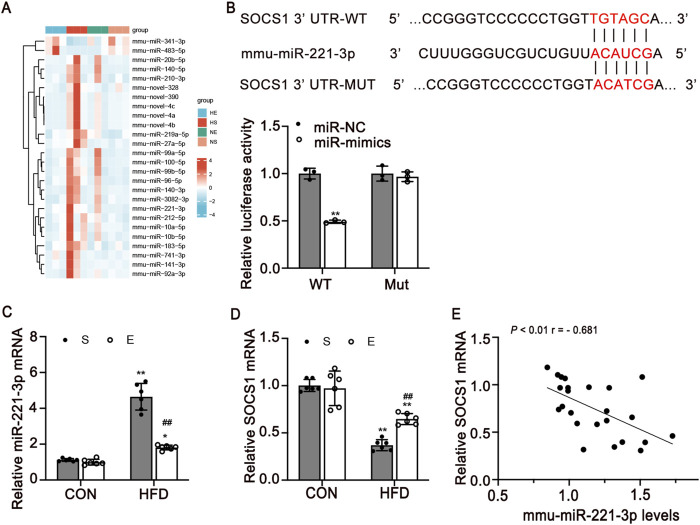
SOCS1 is a direct target of miR-221-3p in skeletal muscle. **(A)** Heatmap of differentially expressed miRNAs in four groups. Legend on the right indicates the log fold change of the genes. **(B)** Dual-luciferase reporter assay identified the target sites between miR-221-3p and SOCS1 3′UTR (n = 3/group). **(C, D)** Expression levels of miR-221-3p and SOCS1 mRNA expression in skeletal muscle (n = 6). **(E)** Pearman correlation between miR-221-3p expression and SOCS1 mRNA expression in skeletal muscle of all mice (r, correlation coefficient). CON, normal control; HFD, high-fat diet; S, sedentary; E, exercise. Values are means ± SD. *P < 0.05, **P < 0.01 vs NS group; ^#^ *P* < 0.05, ^##^ *P* < 0.01 vs HS group.

To identify differentially expressed miRNAs by HFD diet and aerobic exercise treatment, we performed small RNA-sequence in skeletal muscle. 58 microRNAs were differentially expressed from HFD-exercise mice while 59 were differentially expressed from HFD mice in skeletal muscle, including 26 microRNAs that were concordantly regulated in both models (Figure 5A). miR-221-3p is a strong candidate that was increased 1.88-fold and decreased 1.25-fold in the HFD and exercise models, respectively (*P* < 0.001 and *P* = 0.016) (Supplementary Table S1).

To determine the possible mechanism underlying exercise-induced regulation of macrophage polarization, we used algorithms in TargetScan. Our investigation revealed that *Socs1* emerges as a prospective target of miR-221-3p (Figure 5B). We then carried out a dual luciferase reporter assay for miR-221-3p. miR-221-3p overexpression significantly reduced the luciferase activity of the reporter genes containing wild-type 3′-UTR regions of *Socs1*, but no significant changes were observed in the mutated *Socs1* region (Figure 5B).

Furthermore, miR-221-3p and *Socs1* mRNA expression levels in skeletal muscle were detected using quantitative real-time PCR. Notably, an elevation in miR-221-3p levels was observed in the HFD group as compared to the CON group. Intriguingly, the implementation of aerobic exercise led to a significant reduction in miR-221-3p expression (*P* < 0.01, Figure 5C). Conversely, the levels of *Socs1* were found to be attenuated in the skeletal muscle of HFD mice in contrast to the CON group. Remarkably, this reduction was counteracted through engagement in aerobic exercise (*P* < 0.01, Figure 5D). To further validate the negative regulatory role of miR-221 on *Socs1*, a comprehensive analysis of the correlation between miR-221-3p and *Socs1* was undertaken. The outcomes unveiled a negative correlation between the expression levels of *Socs1* and miR-221-3p, with statistical significance (*P* < 0.01, Figure 5E).

### 3.5 Exercise prevented IR by regulating macrophage polarization via SOCS1 and JAK/STAT signaling

**FIGURE 6:**
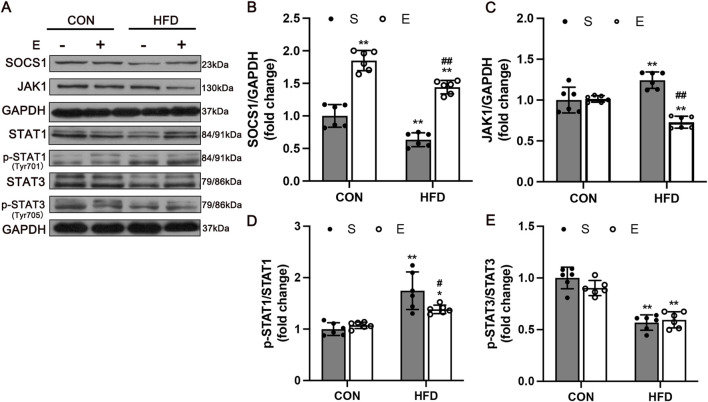
Expression of Macrophage Polarization Related Protein in Skeletal Muscle after Aerobic Exercise Training. **(A)** Representative Western blot images showing a modification of SOCS1-mediated inflammation signaling. GAPDH staining was used as a loading control. Quantification of SOCS1 **(B)**, JAK1 **(C)**, p-STAT1 **(D)**, and p-STAT3 levels **(E)** (n = 6/group). CON, normal control; HFD, high-fat diet; S, sedentary; E, exercise. Values are means ± SD (n = 3/group). *P < 0.05, **P < 0.01 vs NS group; ^#^ *P* < 0.05, ^##^ *P* < 0.01 vs HS group.

To determine whether SOCS1 is involved in regulating M1 and M2 macrophage polarization, we examined the protein levels of SOCS1 and macrophage polarization-related signaling factors (Figure 6A). The results showed that the expression of SOCS1 in HFD mice was significantly decreased compared with the CON group (*P* < 0.01). Aerobic exercise showed a greater increase of SOCS1 protein level compared with the HS group (*P* < 0.01, Figure 6B). The effects of exercise on improving M1 macrophage polarization were detected by measuring JAK1 and the levels of phosphorylated and total STAT1, STAT3. Along with reducing SOCS1 expression, HFD remarkedly elevated JAK1 and p-STAT1 and decreased p-STAT3 levels compared with the CON group (*P* < 0.01, Figures 6C–E). Meanwhile, the expression of JAK1(*P* < 0.01, Figure 6C) and p-STAT1 (*P* < 0.05, Figure 6D) were significantly reduced in the HE mice compared with the HS group.

## 4 Discussion

**FIGURE 7:**
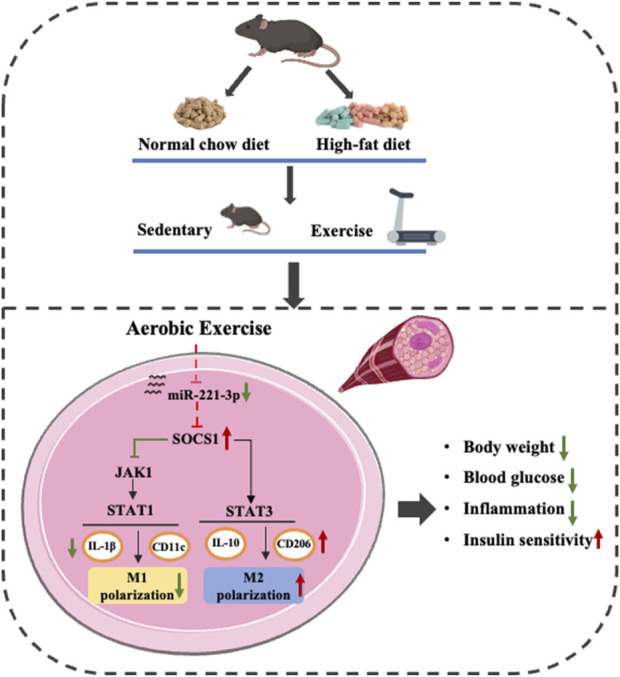
Effect of aerobic exercise on improving chronic inflammation and insulin resistance through miR-221-3p and SOCS1 and regulating macrophage polarization.

In this study, we revealed a novel molecular mechanism through which exercise induces M2 polarization and improves IR. Our investigation unequivocally affirms the following key findings: (i)Aerobic exercise ameliorates weight gain and hyperglycemia caused by HFD. (ii) Aerobic exercise reverses HFD-induced chronic inflammation and IR through macrophage phenotypic polarization. (iii) Aerobic exercise alleviates inflammation by modulating M1 macrophage activation by reducing miR-221-3p expression enhancing the SOCS1 cascade and regulating JAK/STAT signaling (Figure 7). Hence, these results suggest a possible role for miR-221-3p in reciprocal signaling between regulating macrophage polarization and glucose homeostasis, with the potential to induce chronic inflammation and insulin resistance in skeletal muscle.

Obesity-induced IR stands as a recognized risk factor for diabetes, cardiovascular disease, and various cancers. It is well known that regular exercise can improve hyperglycemia and dyslipidemia, which are crucial for delaying the onset of chronic diseases and enhancing quality of life (Di Meo et al., 2017; Li et al., 2021). Our findings demonstrate that aerobic exercise improves glucose uptake, glucose homeostasis, and insulin sensitivity in skeletal muscle, which is consistent with our hypothesis that regular exercise would mitigate the metabolic disruptions caused by obesity-induced insulin resistance. This is in accordance with previous research that has suggested exercise as an effective intervention for improving insulin sensitivity and metabolic function in obese or insulin-resistant models (Cheng et al., 2022; Pillon et al., 2020). Aerobic exercise is found to reduce body weight, particularly by decreasing adipose tissue. The observed increase in liver weight in the NE group is likely due to the chronic effects of exercise. This adaptive response may involve alterations in liver metabolism to meet the increased metabolic demands associated with sustained aerobic exercise. These findings emphasize the importance of regular exercise as a countermeasure to metabolic dysfunction induced by high-fat diets, supporting its role in preventing obesity-related metabolic diseases.

It is well-established that obesity-induced increasing free fatty acids (FFA), influx into skeletal muscle, leading to chronic inflammation (Chazaud, 2020). Similarly, immune cells such as macrophages infiltrate the adipose tissue, liver, and skeletal muscle during obesity, give rise to inflammatory states, and contribute to IR and T2DM (Fujisaka, 2021; Orliaguet et al., 2020; Wu and Ballantyne, 2017). Consistent with previous studies, our results show that high-fat diet-induced mice exhibit a pro-inflammatory state in skeletal muscle, characterized by elevated levels of inflammatory cytokines such as TNF-α and IL-1β, along with activation of inflammatory pathways, which contribute to the progression of IR (Cade, 2018). In contrast, aerobic exercise reduced the levels of IL-1β while increasing IL-10 levels. These findings support the hypothesis that exercise can mitigate inflammation in skeletal muscle. Further investigation revealed that IL-10, as an anti-inflammatory factor, plays a role in macrophage polarization towards the M2 phenotype (Shrivastava and Shukla, 2019). Obesity has been shown to elevate the expression of CD11c in skeletal muscle, which serves as a marker for the M1 phenotype (Fink et al., 2014; Ieronymaki et al., 2019). Our study aligns with these findings, showing significant increases in CD11c+/F4/80+ positive cells in HFD mice, indicating an abundance of M1 macrophages. Our findings also demonstrate that aerobic exercise leads to an increase in CD206+/F4/80+ positive cells and a lower M1/M2 macrophage radio in HFD mice. In hence, our study provides further evidence that exercise-induced changes in macrophage polarization could be a key mechanism by which exercise alleviates chronic inflammation and IR in obesity. However, the precise role of macrophage polarization in skeletal muscle remains incompletely understood.

Currently, miRNAs are being explored as biomarkers in response to physical activity, reflecting their important role in regulating gene expression and metabolic pathways (Soplinska et al., 2020). Regular exercise is beneficial for modulating miRNA expression in skeletal muscle, contributing to improvements in metabolic function and inflammatory regulation (Paschalis et al., 2011). Among these, miR-221-3p has been identified as a key player in M1 macrophage polarization (Wang et al., 2014; Liu et al., 2021). This highlights the relevance of miRNAs as critical regulators in immune and inflammatory processes, especially in the context of obesity-induced metabolic dysfunction. SOCS1 is particularly important in maintaining immune homeostasis by preventing excessive activation of inflammatory signaling. Recent studies have reported that miR-221-3p promotes M1 macrophage activation by directly targeting SOCS1, thereby disrupting this regulatory mechanism (Cai et al., 2020). To validate the relationship between SOCS1 and miR-221-3p, we demonstrated that SOCS1 is a direct target of miR-221-3p and that miR-221-3p negatively regulates SOCS1 expression. Given the role of SOCS1 in balancing inflammatory states, it has also been suggested as a therapeutic target to inhibit the progression of diabetic complications (Recio et al., 2014). These findings are particularly relevant in the context of macrophage polarization, where SOCS1 plays a crucial role in promoting the anti-inflammatory M2 phenotype. Our results further align with previous studies, emphasizing the importance of miR-221-3p/*Socs1* as a potential modulator of macrophage function and a target for mitigating inflammation (Yue et al., 2023; He et al., 2023).

One of the major pathways influenced by miR-221-3p is the Janus kinase (JAK)-signal transducer and activator of transcription (STAT) pathway, which plays a pivotal role in immune regulation and inflammatory responses (Quero et al., 2019). In the present study, we observed a significant elevation in the expression of miR-221-3p in HFD mice, which was accompanied by an increase in M1 macrophage proteins (JAK1 and p-STAT1) and a reduction in M2 macrophage proteins (SOCS1 and p-STAT3). This suggests that the excessive activation of miR-221-3p contributes to chronic inflammation and insulin resistance in skeletal muscle, likely through the dysregulation of SOCS1 and overactivation of the JAK/STAT pathway. Mechanistically, this aligns with the proposed role of miR-221-3p as a pro-inflammatory regulator that disrupts macrophage polarization balance. Moreover, an 8-week aerobic exercise intervention markedly reduced the levels of miR-221-3p, while simultaneously increasing both protein and mRNA levels of S*ocs1*. This was accompanied by a significant reduction in the protein levels of JAK1 and p-STAT1. These results suggest that aerobic exercise can downregulate pro-inflammatory miR-221-3p and restore SOCS1 expression, thereby rebalancing macrophage polarization. This shift towards the anti-inflammatory M2 phenotype not only mitigates chronic inflammation but also improves insulin sensitivity. Although the p-STAT3/STAT3 ratio in skeletal muscle showed no significant difference between the HS and HE groups, exercise selectively activated STAT3 signaling in muscle macrophages, leading to M2-like polarization. Future studies are needed to explore the molecular pathways driving STAT3 activation in muscle macrophages and the role of other immune modulators in this process.

## 5 Conclusion

Overall, our current findings indicate that miR-221-3p emerges as a potential regulator of macrophage polarization. Additionally, aerobic exercise inhibits M1 macrophage polarization through the miR-221-3p/S*ocs1* axis, leading to improvements in both inflammatory states and IR in skeletal muscle. Therefore, aerobic exercise emerges as a promising non-pharmacological intervention for combating insulin resistance and inflammation in skeletal muscle. MiR-221-3p holds promise as a viable target for future strategies aimed at preventing IR.

## Data Availability

The datasets presented in this study can be found in online repositories. The names of the repository/repositories and accession number(s) can be found in the article/Supplementary Material.
