# Supplementary material for: Aerobic exercise improves inflammation and insulin resistance in skeletal muscle by regulating miR-221-3p via JAK/STAT signaling pathway
Source: Front Physiol. 2025 Feb 25;16:1534911. doi: 10.3389/fphys.2025.1534911 (PMC11893602; doi:10.3389/fphys.2025.1534911)
Supplement: Supplementary file 1 [file Table1.docx]

Supplementary Material

**Supplemental Table 1. miRNA levels in miRNA sequencing**

|  | HE-HS | | | HS-NS | | |
| --- | --- | --- | --- | --- | --- | --- |
| miRNAs | regulations | fold change | p-value | regulations | fold change | p-value |
| mmu-miR-100-5p | down | -1.0883137 | 0.01666442 | up | 1.13310788 | 0.00619645 |
| mmu-miR-10a-5p | down | -1.2336295 | 0.01235229 | up | 1.22513534 | 0.00549471 |
| mmu-miR-10b-5p | down | -1.009294 | 0.01797904 | up | 1.22670965 | 0.00176896 |
| mmu-miR-140-5p | down | -2.3590279 | 0.00110456 | up | 2.68865171 | 0.00013129 |
| mmu-miR-140-3p | down | -1.8682302 | 0.00150081 | up | 1.62452233 | 0.00463349 |
| mmu-miR-141-3p | down | -2.7226475 | 0.00104012 | up | 2.6762801 | 0.00063226 |
| mmu-miR-183-5p | down | -1.3018756 | 0.00666313 | up | 1.35505082 | 0.00333355 |
| mmu-miR-20b-5p | down | -2.176209 | 0.01529514 | up | 2.31997054 | 0.00979428 |
| mmu-miR-210-3p | down | -1.3774247 | 0.04362232 | up | 1.66217309 | 0.01991467 |
| mmu-miR-212-5p | down | -1.2292235 | 0.01576159 | up | 1.46184838 | 0.00340604 |
| mmu-miR-219a-5p | down | -1.6289258 | 0.04237259 | up | 1.94641851 | 0.02333198 |
| mmu-miR-221-3p | down | -1.2458281 | 0.01695229 | up | 1.88283989 | 9.7987E-05 |
| mmu-miR-27a-5p | down | -1.5521383 | 0.01630951 | up | 1.94170901 | 0.00282196 |
| mmu-miR-3082-3p | down | -2.0179286 | 0.00684311 | up | 1.51712431 | 0.02402629 |
| mmu-miR-341-3p | up | 2.18182555 | 0.00945505 | down | -2.2680262 | 0.00912347 |
| mmu-miR-483-5p | up | 4.80396022 | 0.01016085 | down | -4.1732669 | 0.02971403 |
| mmu-miR-741-3p | down | -2.2479806 | 0.03988453 | up | 2.71107503 | 0.00907972 |
| mmu-miR-92a-3p | down | -1.5718485 | 0.00287091 | up | 1.19562425 | 0.01946867 |
| mmu-miR-96-5p | down | -1.9252365 | 0.00161134 | up | 2.48230897 | 2.8396E-06 |
| mmu-miR-99a-5p | down | -1.5620109 | 0.002463 | up | 1.75136373 | 0.00038219 |
| mmu-miR-99b-5p | down | -1.3102575 | 0.00151364 | up | 1.48288579 | 0.0001364 |
| mmu-novel-328 | down | -2.0238156 | 0.00615804 | up | 1.44940834 | 0.04112434 |
| mmu-novel-390 | down | -4.107474 | 0.00000213 | up | 2.8613622 | 0.00035715 |
| mmu-novel-4a | down | -3.896333 | 3.21E-07 | up | 2.65512542 | 0.00031686 |
| mmu-novel-4b | down | -3.896333 | 3.21E-07 | up | 2.65512542 | 0.00031686 |
| mmu-novel-4c | down | -3.896333 | 3.21E-07 | up | 2.65512542 | 0.00031686 |
