# Supplementary figures and images for: Aerobic exercise improves inflammation and insulin resistance in skeletal muscle by regulating miR-221-3p via JAK/STAT signaling pathway
Source: Front Physiol. 2025 Feb 25;16:1534911. doi: 10.3389/fphys.2025.1534911 (PMC11893602; doi:10.3389/fphys.2025.1534911)

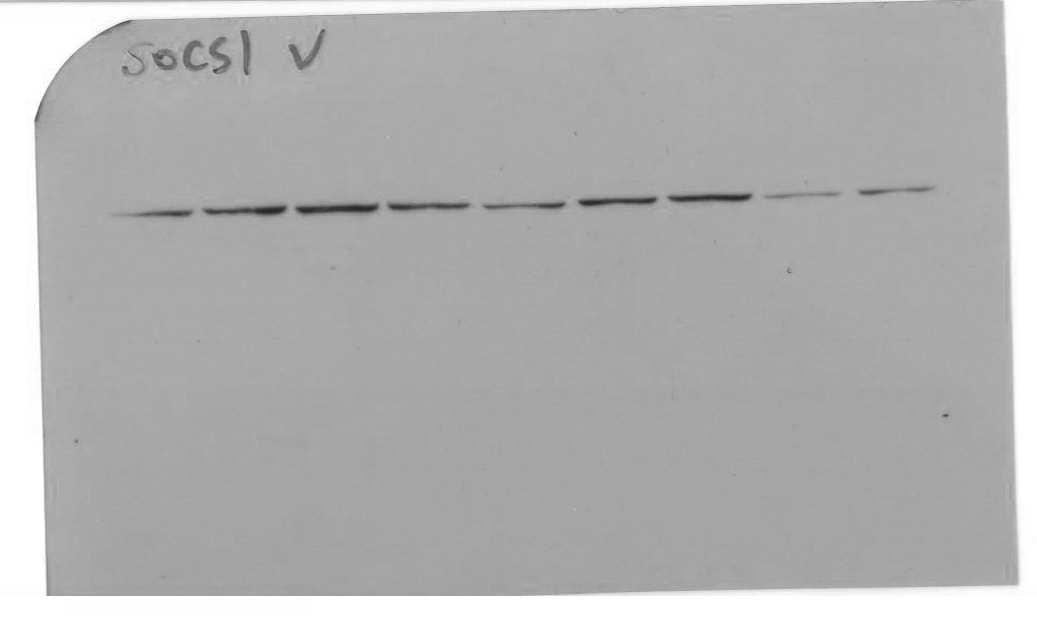

Supplement: Supplementary file 2 [file DataSheet1.zip › GEL/SOCS1.tif]

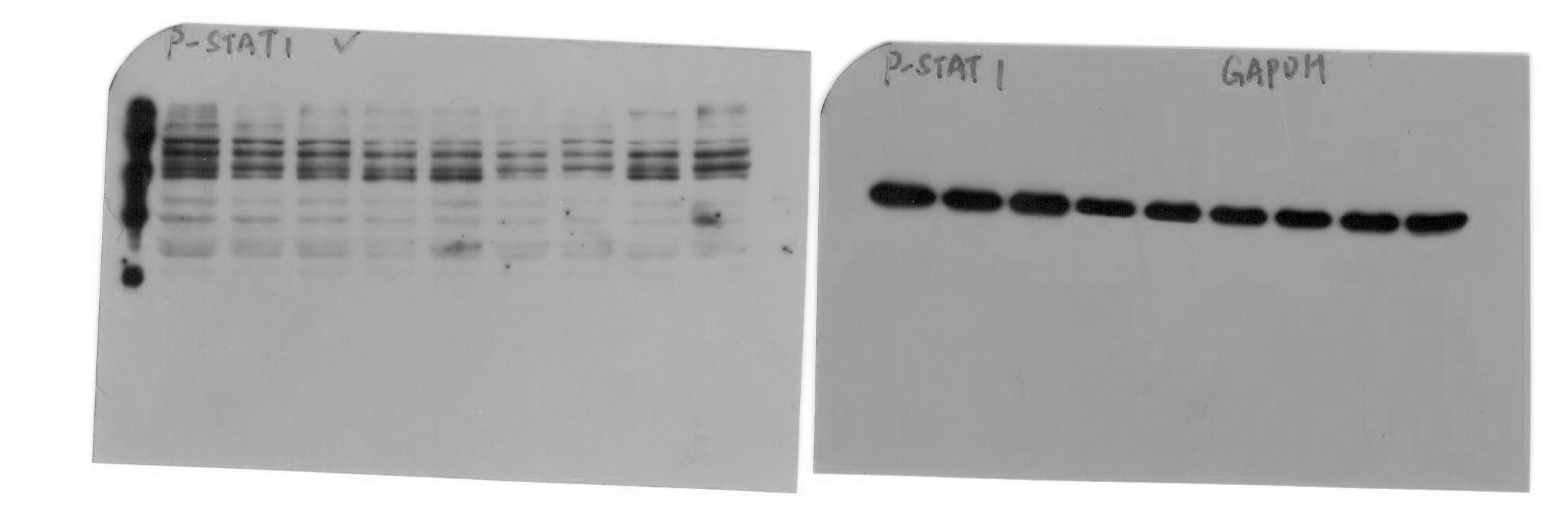

Supplement: Supplementary file 2 [file DataSheet1.zip › GEL/p-STAT1+GAPDH.tif]

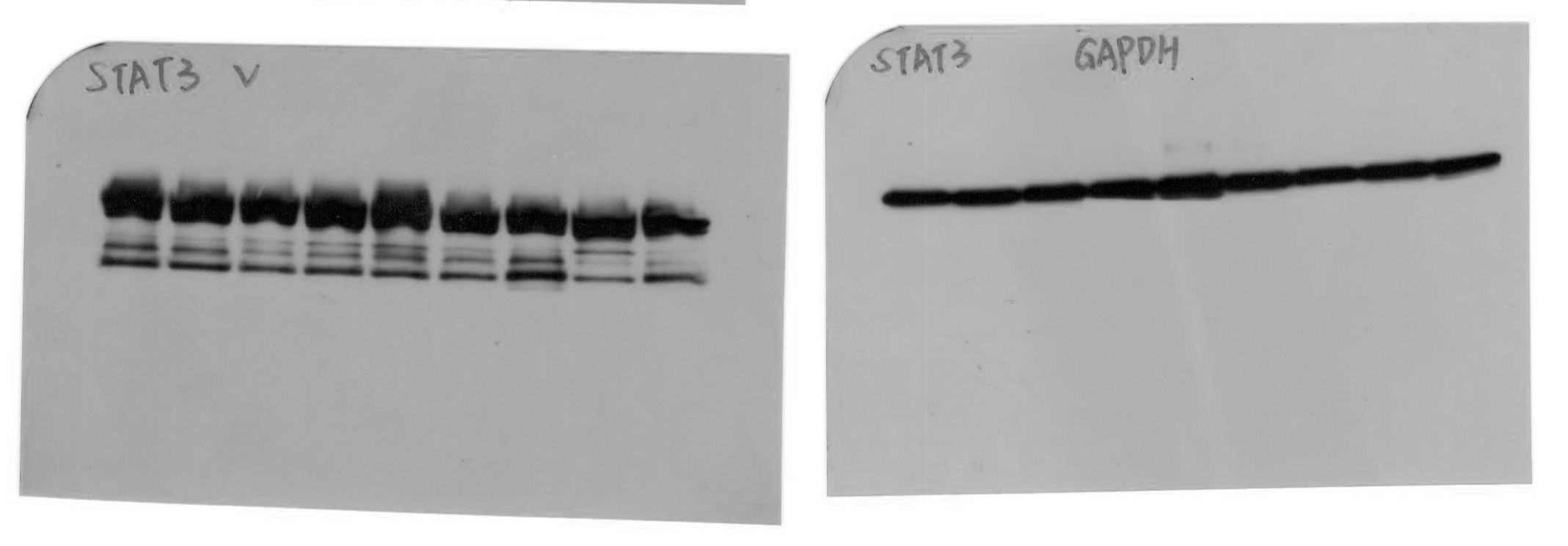

Supplement: Supplementary file 2 [file DataSheet1.zip › GEL/STAT3+GAPDH.tif]

## SOCS1

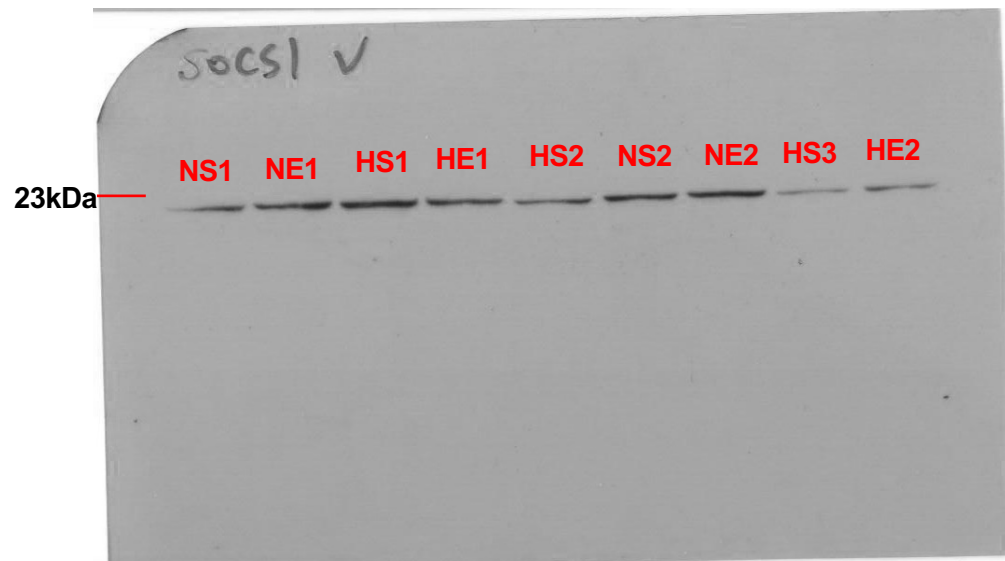

## GAPDH

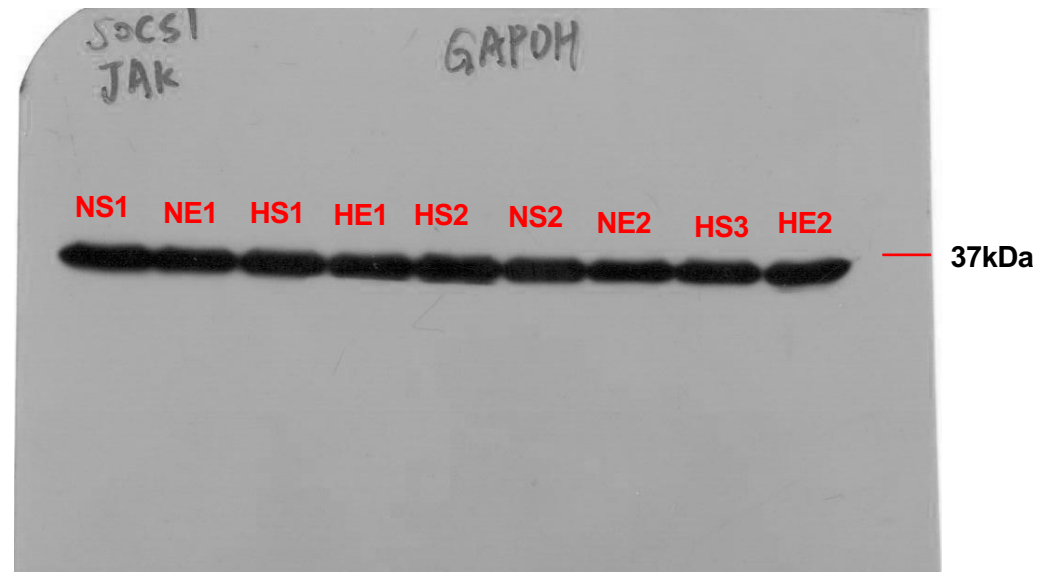

## JAK1

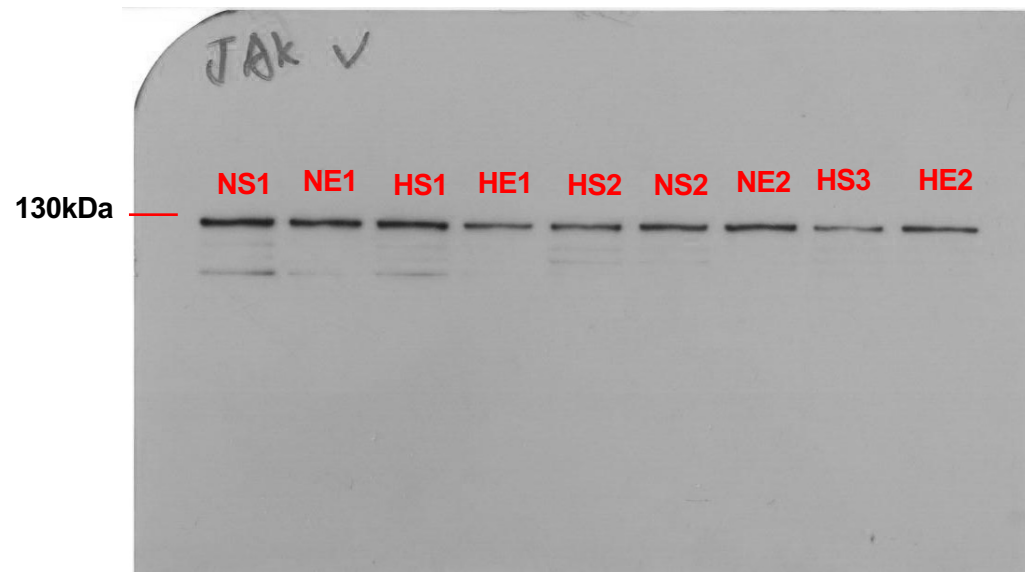

## GAPDH

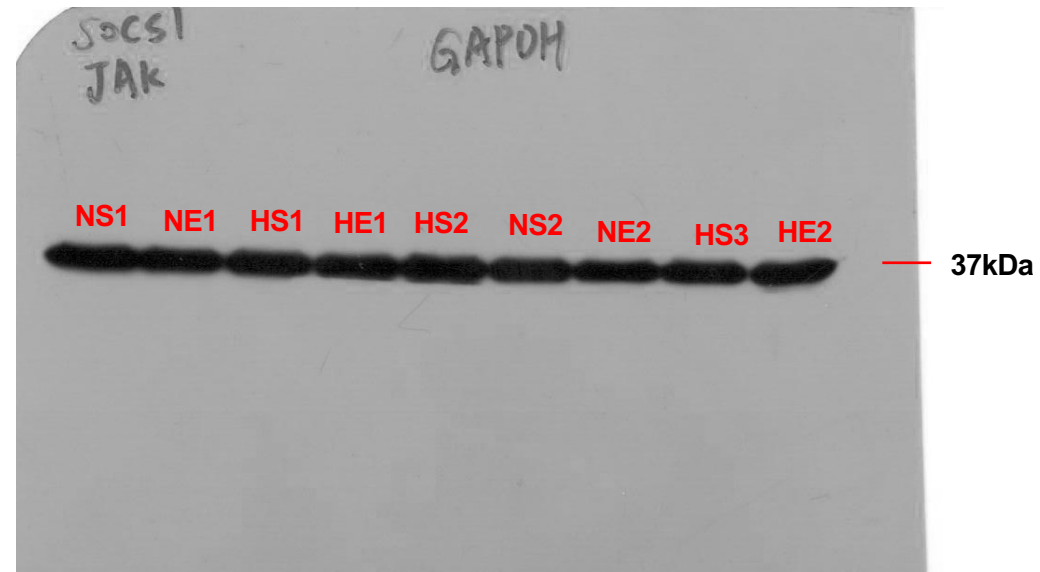

STAT1

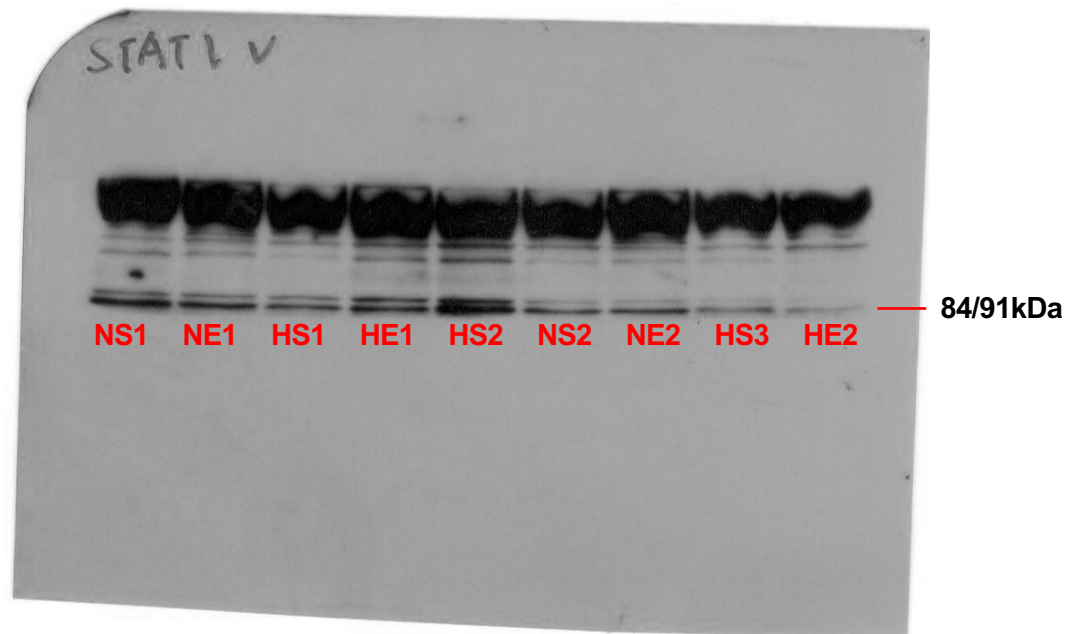

p-STAT1

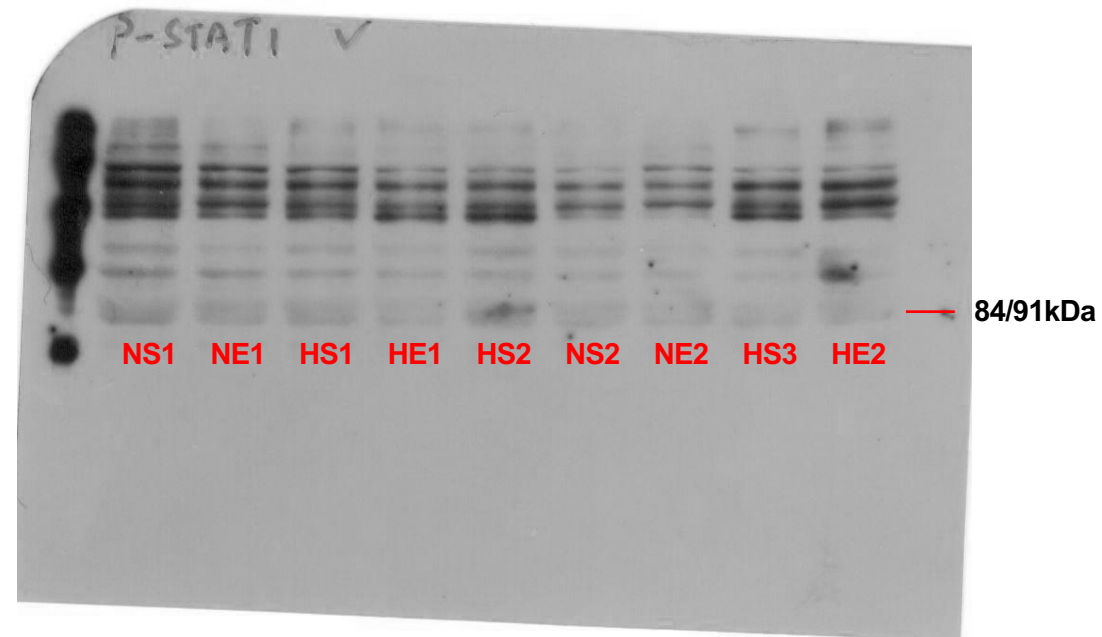

GAPDH

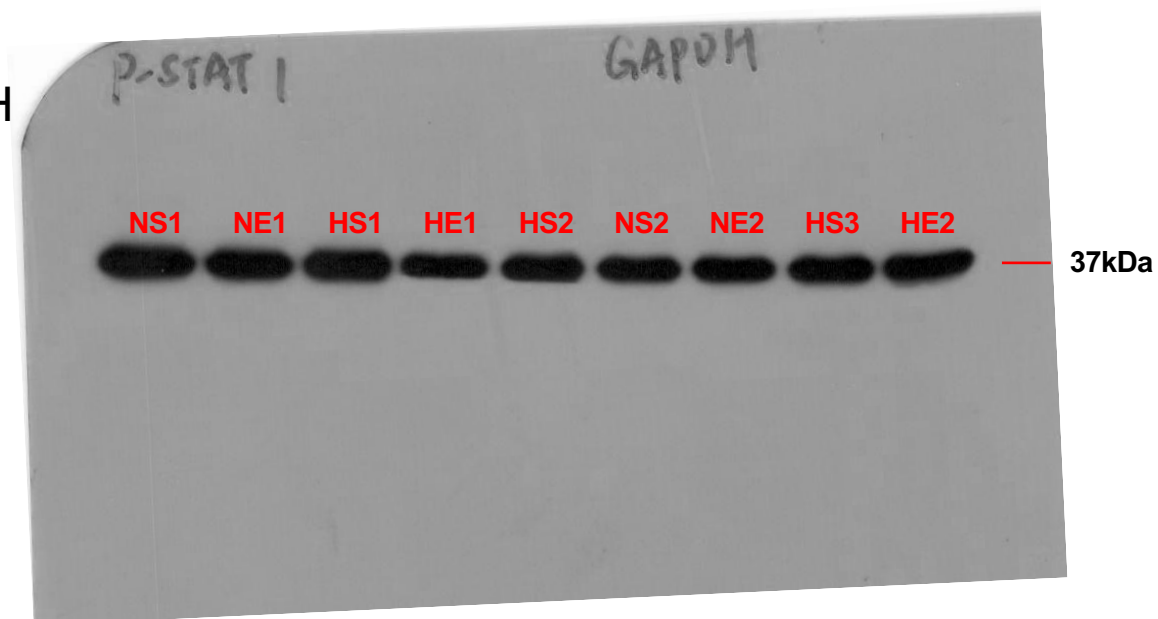

STAT3

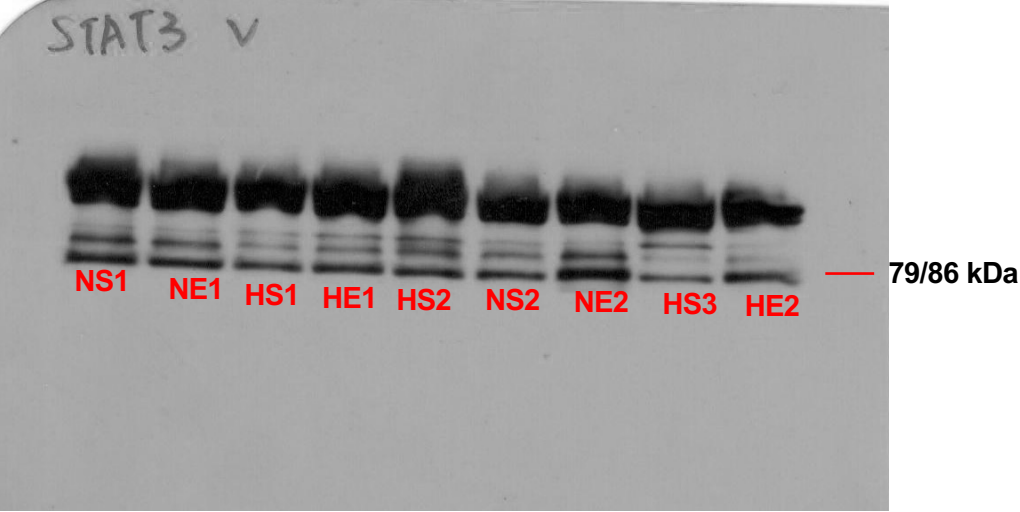

p-STAT3

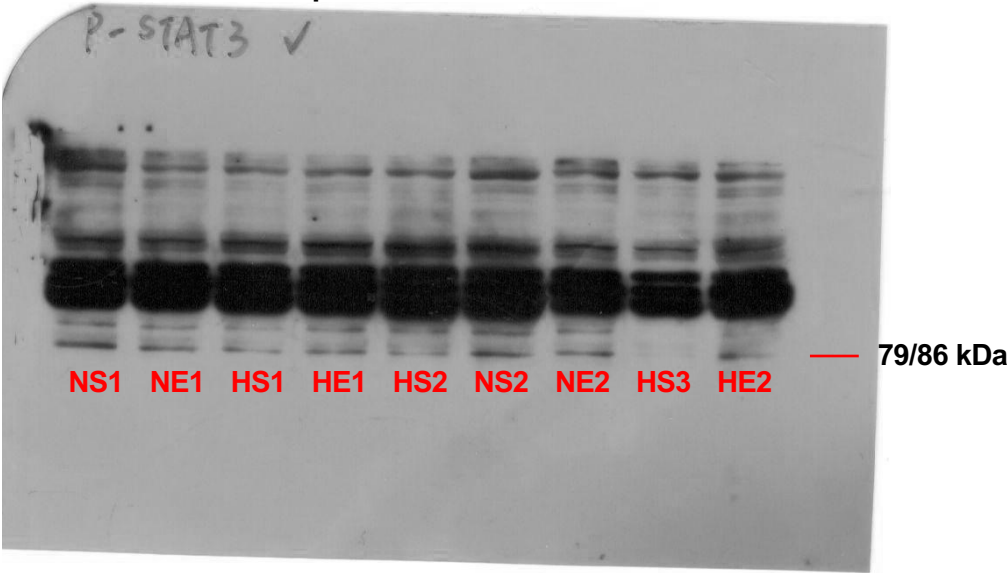

GAPDH

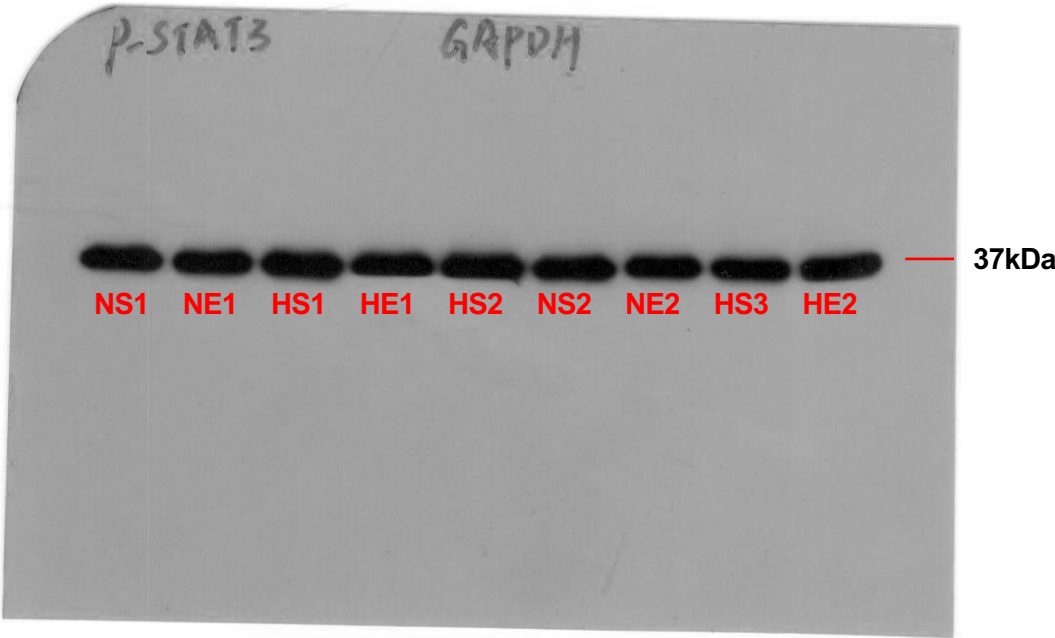

Supplement: Supplementary file 2 [file DataSheet1.zip › GEL/Original Images for Blots and Gels.pdf]

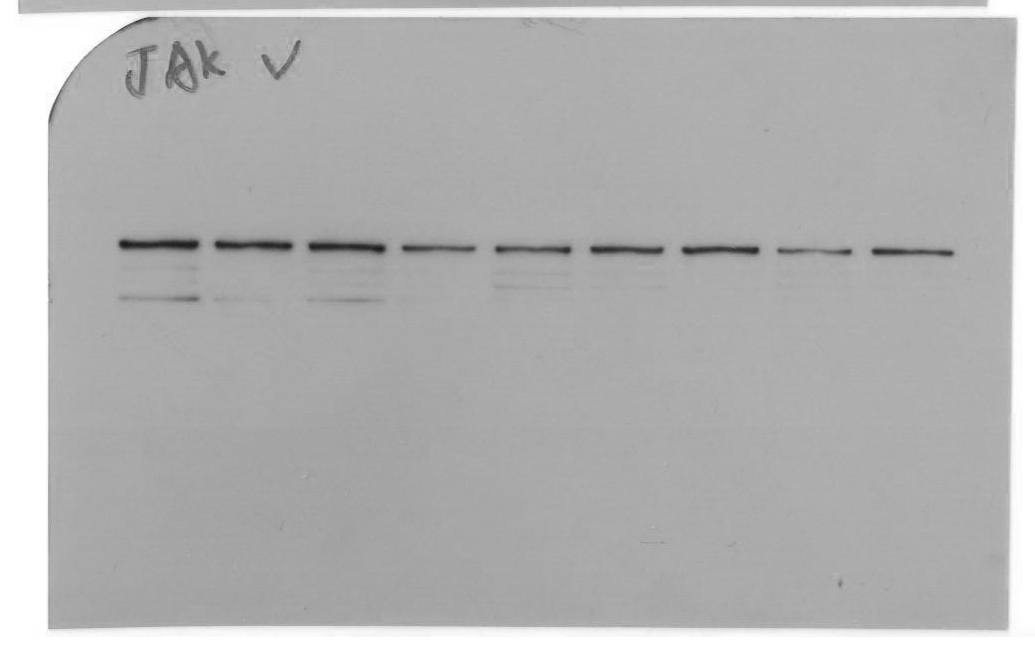

Supplement: Supplementary file 2 [file DataSheet1.zip › GEL/JAK1.tif]

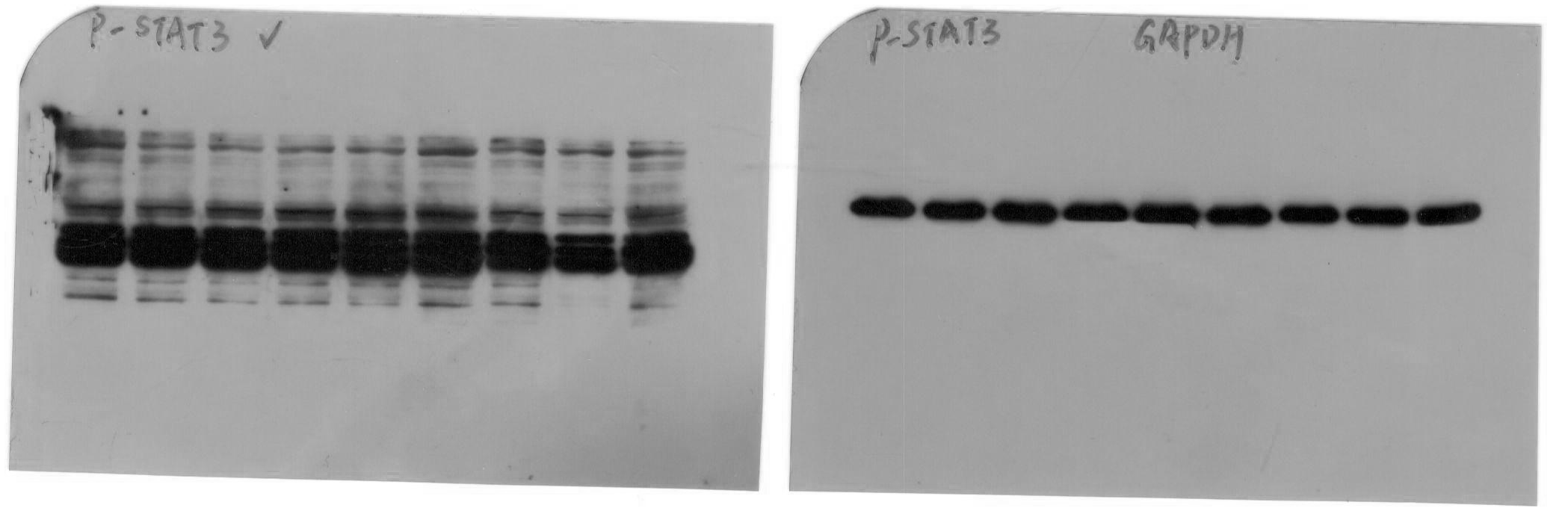

Supplement: Supplementary file 2 [file DataSheet1.zip › GEL/p-STAT3+GAPDH.tif]

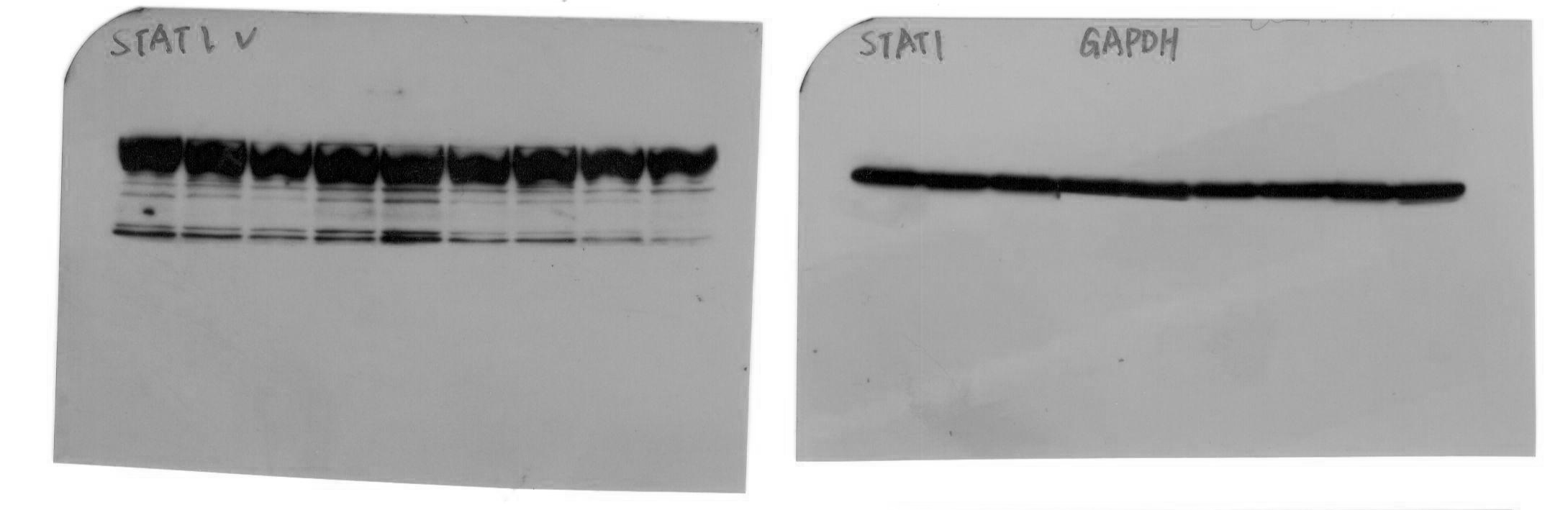

Supplement: Supplementary file 2 [file DataSheet1.zip › GEL/STAT1+GAPDH.tif]
